# Malignant hemopathy and COVID-19: one year later, lessons from the COVID-19 pandemic

**DOI:** 10.11604/pamj.2022.41.63.29705

**Published:** 2022-01-24

**Authors:** Mounia Bendari

**Affiliations:** 1 Mohammed VI University of Health Sciences, Casablanca, Morocco

**Keywords:** COVID-19, malignant hemopathy, lessons

## To the Editors of the Pan African Medical Journal

In late December 2019 an outbreak of a novel coronavirus (SARS-CoV-2) causing severe pneumonia (COVID-19) was reported in China. Patients infected with SARS-CoV-2 can have acute respiratory distress syndrome, with a high likelihood of admission to intensive care, and probably die [1,2]. On the basis of studies conducted in China and elsewhere, many biological markers was important and their value as prognostic indicators have been established [3].

The patients followed for malignant hemopathies are particularly fragile and predisposed to infections indeed since the start of the pandemic. On our unit, we have redoubled our vigilance and we had to adapt several intensive chemotherapy protocols and use more growth factors to reduce the post-chemotherapy aplastic phase. Early on the pandemic, we suspended the activity of bone marrow transplants (both autografts and allografts), we followed the local recommendations of the Moroccan society of hematology inspired by the recommendations issued by international learned societies (ASH, SFH, EHA, EBMT, SFGM-TC, IFM) which are often adapted to our context.

Chemotherapy doses were changed to reduce clinical neutropenia without compromising the curative potential of treatment; patients were isolated in separate rooms for security reasons. Autologous stem cell transplants were postponed for all patients, replaced by cryopreservation grafts already mobilized, the decision was made by a multidisciplinary team until the risks associated with the COVID-19 pandemic had passed. COVID-19 had an impact on the availability of labile blood products and the availability of voluntary donors which was due to confinement. We proceeded by a program established in advance to ensure supply of the blood bank by creating awareness of families of aplastic patients, special authorization for blood donation, and an apheresis platelet program has been implemented.

One year after start of SARS-CoV-2 pandemic, our activity became normal, in Morocco, the situation is less critical than in Europe and the United States. In fact, activity of bone marrow transplants, both autografts and allografts restarted. Among patients followed for hematological malignancies, 23 patients were infected by COVID-19, 6 patients were treated for aggressive lymphomas, 6 cases of multiple myeloma, 5 myeloid acute leukemia, 2 lymphoblastic lymphoma, 2 myelodysplastic syndromes; for patients with acute leukemia they were on aplastic phase. Contrary to our forecasts, those particularly fragile patients presented a minor form of the infection of COVID-19 with good progress for the majority of them. Deaths due to the evolution of the neoplasia are reported for 3 patients followed for multiple myeloma. No death secondary to the COVID-19 infection was noted.

Our preliminary results remain limited and more investigation is required to study the particularity of those patients and identify the real risk factor of mortality for those patients. Our personal and professional lives have been deeply impacted by the COVID-19 pandemic. However, it must be recognized that this crisis also has advantages, in particular the rigor in terms of hospital hygiene. The vaccination plan is well carried out in our country. However, we must remain vigilant in the face of the emergence of new strains of the virus.
